# Valorization of Apple Peels through the Study of the Effects on the Amyloid Aggregation Process of κ-Casein

**DOI:** 10.3390/molecules26082371

**Published:** 2021-04-19

**Authors:** Valeria Guarrasi, Giacoma Cinzia Rappa, Maria Assunta Costa, Fabio Librizzi, Marco Raimondo, Vita Di Stefano, Maria Antonietta Germanà, Silvia Vilasi

**Affiliations:** 1 Istituto di Biofisica, Consiglio Nazionale delle Ricerche, Via Ugo La Malfa 153, 90146 Palermo, Italy; cinziarp@hotmail.it (G.C.R.); mariaassunta.costa@cnr.it (M.A.C.); fabio.librizzi@cnr.it (F.L.); raimondo.marco@gmail.com (M.R.); silvia.vilasi@cnr.it (S.V.); 2 Dipartimento Scienze e Tecnologie Biologiche Chimiche e Farmaceutiche, Università degli Studi di Palermo, Via Archirafi 32, 90123 Palermo, Italy; vita.distefano@unipa.it; 3 Dipartimento di Scienze Agrarie, Alimentari e Forestali, Università degli Studi di Palermo, Viale delle Scienze Ed. 4, 90128 Palermo, Italy; mariaantonietta.germana@unipa.it

**Keywords:** polyphenolic extract, fruit waste, κ-casein amyloid aggregation

## Abstract

Waste valorization represents one of the main social challenges when promoting a circular economy and environmental sustainability. Here, we evaluated the effect of the polyphenols extracted from apple peels, normally disposed of as waste, on the amyloid aggregation process of κ-casein from bovine milk, a well-used amyloidogenic model system. The effect of the apple peel extract on protein aggregation was examined using a thioflavin T fluorescence assay, Congo red binding assay, circular dichroism, light scattering, and atomic force microscopy. We found that the phenolic extract from the peel of apples of the cultivar “Fuji”, cultivated in Sicily (Caltavuturo, Italy), inhibited κ-casein fibril formation in a dose-dependent way. In particular, we found that the extract significantly reduced the protein aggregation rate and inhibited the secondary structure reorganization that accompanies κ-casein amyloid formation. Protein-aggregated species resulting from the incubation of κ-casein in the presence of polyphenols under amyloid aggregation conditions were reduced in number and different in morphology.

## 1. Introduction

The recycling and utilization of agricultural wastes is considered to be an important step in environmental sustainability and agricultural development as well as in promoting a circular economy [1]. Large amounts of residue with high levels of organic matter cause serious environmental pollution if discarded or disposed of improperly [2].

In this regard, an important opportunity is provided by the valorization and recycling of peels from apples, which are a rich source of bioactive compounds, like polyphenols [3]. In fact, similar to the whole fruit, the apple peel polyphenol content includes abundant amounts of flavan-3-ols/procyanidines, phenolic acids, flavonols, dihydrochalcones, and anthocyanins [4], known for their beneficial effects in human health [5]. There is evidence that apple polyphenols are strong antioxidants [6] and have, among other benefits, anti-tumor [7], anti-allergy [8], and life-extending properties [9].

Extensive research demonstrated how the polyphenols present in apples are highly promising as potential therapeutics for disorders resulting from protein amyloid aggregation, the process at the basis of severe neurodegenerative diseases, like Parkinson’s, Alzheimer’s (AD), and Huntington’s disease [10,11,12,13]. These pathologies are characterized by the conversion of a normally soluble protein into an insoluble aggregated form, with further deposition onto a cross-β fibrillary structure [14,15,16,17]. A crucial feature of amyloidosis is the complex polymorphism of the species formed whose ultrastructure and morphology are strictly correlated to their specific toxic potentiality [18,19]. The specific low-molecular-weight oligomers that form during the intermediate steps of the process are thought to play a crucial role in interacting with cell membranes and producing reactive oxygen species (ROS), thus causing cellular function impairment that ultimately leads to the death of the involved cells [20,21,22]. Thereby, the polyphenols known for their marked antioxidant properties are very effective at fighting the oxidative stress associated with amyloid toxicity [23,24,25].

An increasing number of studies have demonstrated how several specific polyphenols present in apples, including quercetin, procyanidins, (−)-epigallocatechin-3-gallate (EGCG), (+)-catechin and (−)-epicatechin, ferulic acid, and vanillin, possess the proper structural features to directly interfere with the proteins involved in the amyloid aggregation so as to inhibit their pathological assembly [26,27,28,29,30,31,32,33]. In this regard, a protein that has recently been used as a model system for characterizing the direct interference of polyphenols with proteins susceptible to amyloid formation is κ-casein from bovine milk, which is able to form amyloid fibrillar structures in vitro under physiological conditions (37 °C and neutral pH) [28,34,35].

This is the smallest of the caseins and is responsible for the steric stability of the casein micelles [36]. In native environments, κ-casein forms multimeric colloidal systems through several types of interactions, such as disulfide bonds, electrostatic, hydrogen bonds, and hydrophobic interactions [37,38,39,40]. Researchers observed that κ-casein, both in its native and reduced-carboxymethylated form, is able to form amyloid fibrils in vitro if incubated at 37 °C [41,42,43,44]. The aggregation in vivo is prevented by the activity of the other two caseins, β- and αs1-casein, which are also naturally present in bovine milk [43,44] and known for their chaperone-like activity [45,46,47,48,49]. When the chaperone-system fails, κ-casein forms corpora amylacea in the bovine mammary glands and in milk [45]. Differently from a typical nucleation-polymerization scheme, the rate-limiting step involved in κ-casein amyloid fibrillogenesis is not an initial nuclei formation but the dissociation of amyloidogenic monomers from the initial multimeric state [41,42,50]. This amyloid precursor has a native β-strand conformation with the proper structural features leading to fibrillar aggregation and it has the same biophysical features of the amyloid core formed by Aβ peptide in Alzheimer’s disease [41,42,50]. Similar to the Aβ peptide, κ-casein is the archetype of an amyloidogenic protein belonging to the class of Intrinsically Disordered Proteins (IDPs) [51]. More importantly, native κ-casein forms micelle-like complexes and studies demonstrated that aggregates with micellar properties could play a role in amyloid formation of several proteins [52,53,54]. Studying the effect of some selected compounds on amyloid aggregation of proteins forming micelles could help to individuate inhibitors with a specific role towards such micelle-like conformations also gaining insight into their mechanism of action. For all these reasons, bovine milk protein κ-casein was used to study the effect of specific polyphenols, many of which present in apples, on the amyloid fibril formation mechanism [28,34,35].

In this study, κ-casein was used as a protein model to investigate the effect of polyphenolic extracts from apple peels in the amyloid aggregation processes. The apple cultivar was Fuji grown in Sicily. While several studies concerned the effect on amyloids of individual polyphenols found in apples, only an experiment, carried out by thioflavin T (ThT) assay, focused on the ability of the whole polyphenolic extract obtained from apples to inhibit an amyloid aggregation process [31]. However, the molecular mechanism underlying this inhibitory action is far from understood. This is a critical point to evaluate the therapeutic potentiality of fruit and vegetable total phenolic extracts, already tested for important conditions regarding human health [55], in the field of amyloid diseases.

To the best of our knowledge, no study has been performed on extracts from a waste product, like apple peel, in the amyloid aggregation process. These investigations, aimed at agricultural and food industry waste valorization, offer important incentives in the recovery and reutilization of valuable materials, thus sustaining the eco-system health and circular economy strategies. In fact, the waste from apples used for commercial products, like jam, juice, and jelly, constitute about 30% of the original fruits. This large amount of organic residue seriously contributes to increasing air pollution [2].

Here, we investigated the influence of the apple peel polyphenol extracts on the amyloid assembly process of κ-casein by incubating them with the protein before thermally inducing fibrillization. We used light scattering (LS) and thioflavin T (ThT) fluorescence assay to monitor the effect of the extracts on the κ-casein aggregation, and fibril formation kinetics. Using Congo red binding assay, circular dichroism (CD), and atomic force microscopy (AFM), we monitored the structural and morphological features of the κ-casein species formed in the presence of the apple peel polyphenols (APP).

Our results show that the APP extract inhibited, in a dose-dependent manner, κ-casein amyloid fibril formation, and the structural reorganization that occurs during the protein assembly was inhibited in the presence of APP. The aggregates resulting from κ-casein incubation with APP under amyloid formation conditions were different in number and morphology.

## 2. Results

### 2.1. Total Phenolic Content (TPC), Antioxidant Properties, and Phenolic Characterization of Extracts from Apples Peels

As a prior step to the experiments, we conducted a quantitative determination of the apple peel extract phenolic compounds using the Folin–Ciocalteu method. The results are reported in Table 1 where the TPC is expressed as catechin equivalents. For comparison, in the same table, TPC values from the same apple variety other tissues (flesh and whole fruit) are also reported. The results show that the peel had a significantly higher TPC value over both the flesh and whole fruit.

We also evaluated the antioxidant capacity of the three tissues (peel, whole, and flesh) by using an oxygen radical absorbance capacity (ORAC) assay (Table 1). The ORAC method uses a biologically relevant free radical source (peroxyl radical), which is the most prevalent free radical in human biology [56]. Therefore, ORAC results can be correlated to in vivo studies on the antioxidant activity shown by apple polyphenols [57,58,59,60,61].

As shown in the table, the antioxidant activity of the polyphenolic extract from apple peel was significantly more marked when compared to the other tissues of the fruit.

The polyphenolic profile of Fuji apple peel extract was studied using ultra-high-performance liquid chromatography with heated electrospray ionization coupled to mass spectrometry (UHPLC–HESI–MS) techniques, and seven phenolic compounds were identified (Supplementary Materials, Figure S1). In good agreement with the literature data [5], the qualitative profile showed vanillin, gentisic acid, sinapinic acid, ferulic acid, epicatechin, quercetin, and procyanidin A2.

### 2.2. Ability of the APP Extract to Influence κ-Casein Fibrillogenesis and Disaggregate κ-casein Preformed Fibrils

The effect of polyphenolic extracts from the apple peel on the kinetics of κ-casein aggregation was analyzed by Thioflavin T (ThT) fluorescence assay. ThT is a fluorescent dye that is known to reliably detect the formation of amyloid fibrils due to the significant increase in its emission intensity upon binding to the linear array of β-strands [62,63].

We analyzed the effect of APP at the concentrations of 5, 10, 20, and 40 µg/mL, after testing their toxicity on NHI-3T3 cells. This is an easy-to-handle cell line that immortalized spontaneously [64] and, for this reason, it is considered more representative of normal cells than a tumor cell line. Hence, NIH/3T3 is one of the most frequently used lines in material/cell interaction research, and it has been previously used for studying the toxic effect induced on cells by amyloid insults [22,65,66].

**Figure 1:**
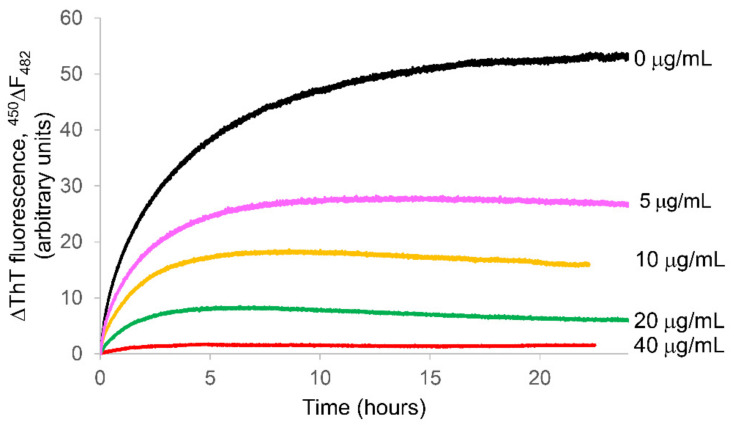
In situ real-time thioflavin T fluorescence assay for monitoring the aggregation kinetics of 50 μM (~1 mg/mL) κ-casein incubated at 37 °C in the absence (black) and in the presence of different APP concentrations: 5 μg/mL (pink), 10 μg/mL (yellow), 20 μg/mL (green), and 40 μg/mL (red).

The above-mentioned concentrations were not cytotoxic after 72 h of treatment as reported in Figure S2 (Supplementary Materials). Figure 1 shows the ThT signal during the fibrillization kinetics of 50 µM κ-casein incubated at 37 °C in the absence and in the presence of different APP concentrations. In the absence of polyphenols, ThT from κ-casein exhibited an increase in fluorescence emission, which is indicative of amyloid formation. The absence of a lag phase in the kinetics profile is due to an aggregation mechanism in which the rate-limiting step is not the initial nuclei aggregation but the dissolution of native protein multimers with subsequent exposition to the solvent of buried amyloidogenic regions [41,42].

In the presence of APP, both the aggregation rate and the fluorescence plateau, corresponding to the total amount of fibrils, were reduced in a concentration dependent manner (Figure 1).

**Figure 2:**
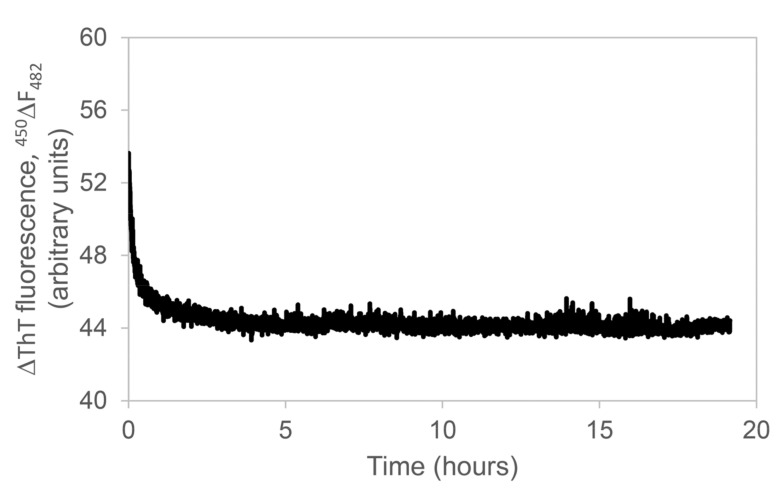
Fluorescence of Thioflavin T as a function of time after the addition of 40 μg/mL APP to 50 μM (~1 mg/mL) κ-casein species formed after 24 h incubation at 37 °C.

As the next step, we verified that, in addition to inhibiting protein aggregation, APP were also able to disaggregate preformed κ-casein amyloid structures. To do this, we added APP at 40 μg/mL to the 50 μM κ-casein sample taken at the end of the aggregation kinetics (after about 24 h at 37 °C) and followed the dye fluorescence for the next 8 h at 37 °C. After an initial phase taken for the system to achieve the thermal equilibration, the fluorescence did not vary in time (Figure 2), thus revealing that, while APP were able to inhibit protein aggregation, they could not disrupt already formed amyloid assemblies.

This experiment was also useful in order to exclude the occurrence of potential interference between the dye and APP. Caution is recommended in the use of thioflavin T assay when probing anti-amyloidogenic compounds that, even if not spectroscopically active at ThT wavelengths, like the APP under analysis (data not shown), can interfere with the dye fluorescence causing a bias affecting the experimental data interpretation [35,67]. Our experiment showed that APP did not cause ThT fluorescence reduction by interfering with the dye in a time of about 8 h, thus excluding the occurring of fluorescence quenching, in this time, by the introduction of phenols.

### 2.3. β-Sheet-Amyloid Formation Assessed by the Congo Red Binding Assay

**Figure 3:**
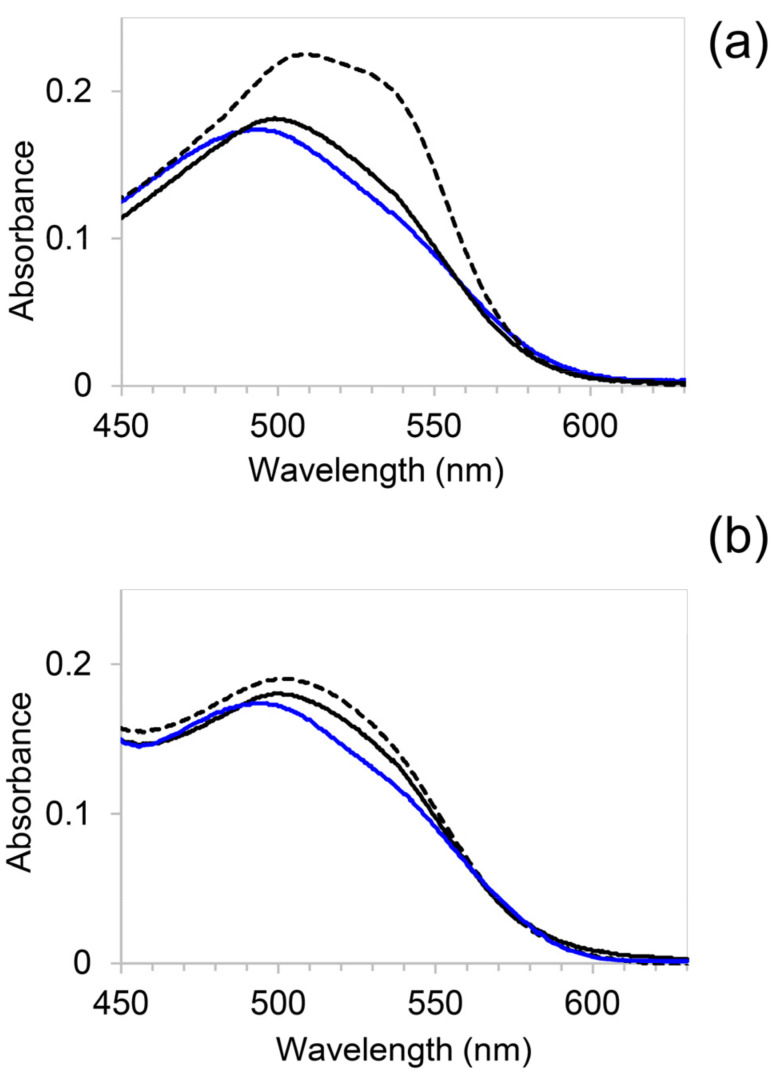
(**a**) Absorption spectra of 20 μM Congo red (blue), Congo red with 50 μM fresh native κ-casein (solid black line), and with 50 μM κ-casein incubated for 24 h at 37 °C (dashed black line). (**b**) Absorption spectra of 20 μM Congo red + 40 μg/mL APP (blue), CR with 50 μM κ-casein + 40 μg/mL APP before (solid black line), and after incubation 24 h at 37 °C (dashed lack line).

We used other experimental tools to confirm the inhibitory APP effect found by the ThT assay. First, we used the absorptive dye, Congo red (CR), which has been demonstrated as a viable alternative to ThT in the case of potential interference with exogenous compounds precisely tested with κ-casein [35]. CR allows the detection of the presence of amyloid β-pleated sheets, because, when bound to β-sheet-rich amyloid fibrils, the CR molecules adopt a specific orientation with their long axis lying parallel to the fibril axis. This causes a molecular CR torsional restriction that induces a characteristic increase in absorption and a red shift in the absorption maximum from 490 to 540 nm [68]. To further investigate inside the β-sheet-amyloid formation in κ-casein aggregates and evaluate the effect of APP on κ-casein assembly process, we added 20 μM CR to aliquots of 50 μM fresh native κ-casein and the protein after 24 h of incubation at 37 °C. The experiment was performed on samples incubated both in the presence and absence of APP at 40 μg/mL. After the dye addition, the CR absorption band was recorded immediately. In Figure 3a, the spectra of CR alone, CR in the presence of fresh native κ-casein, and CR in the presence of κ-casein after 24 h of incubation at 37 °C are presented. In the presence of fresh native κ-casein, the CR spectrum showed, in comparison to free dye, a slight shift from 500 to 510 nm likely due to the dye binding to protein initial micelles. However, a more marked change occurred when CR was incubated with the κ-casein preformed structure formed after 24 h at 37 °C. A shoulder at 540 nm appeared, indicative of CR binding to β-sheet-rich amyloid fibrils [68]. In the presence of polyphenols, no significant change in the absorbance maximum wavelength was observed between the spectra of CR added to the native κ-casein + APP and the same sample taken after 24 h incubation at 37 °C (Figure 3b). This further confirms the APP inhibitory action toward amyloid formation.

### 2.4. Apple Peel Polyphenols Influence on κ-Casein Secondary Structure

**Figure 4:**
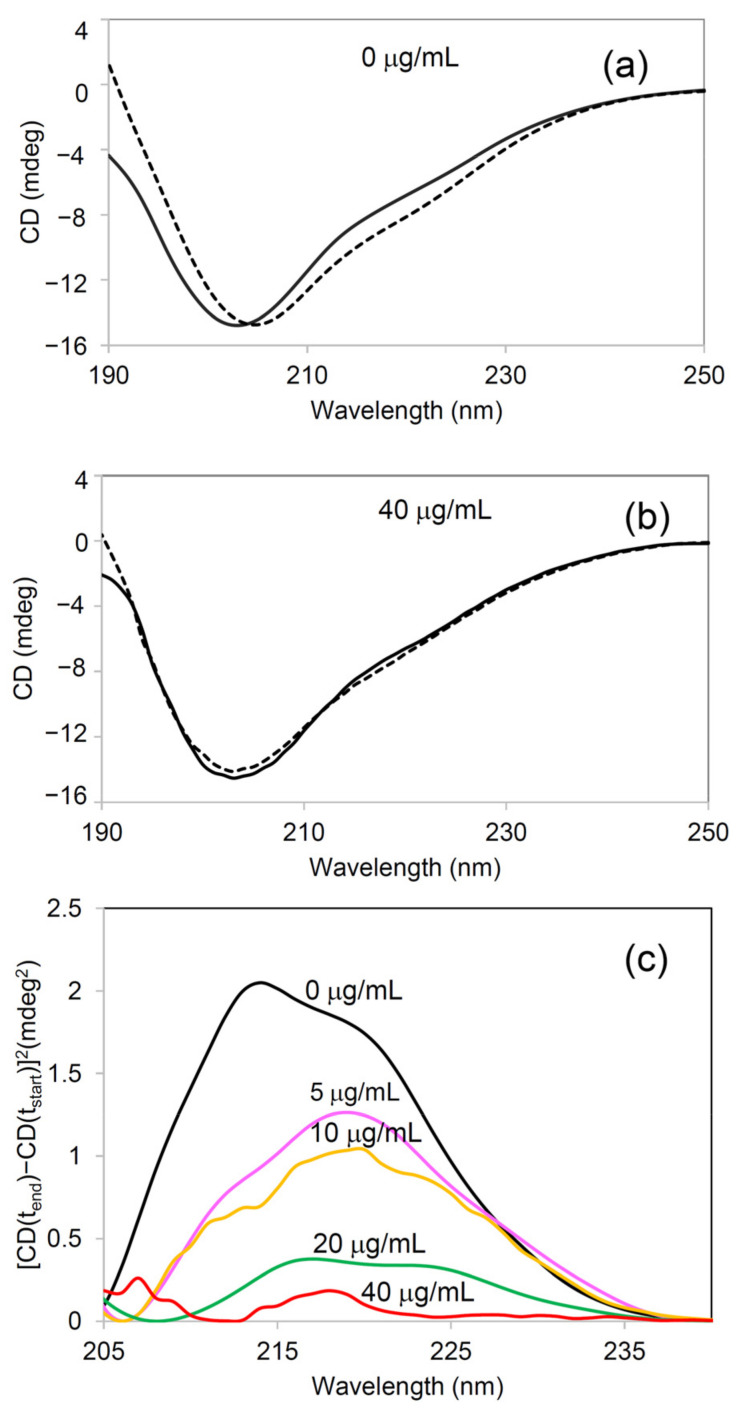
Circular dichroism (CD) spectra of 50 µM κ-casein at the beginning (t_start_) of the amyloid aggregation process (solid black) and after 24 h (t_end_) of incubation at 37 °C (dashed black) in the absence (**a**) and presence of 40 µg/mL of APP (**b**). (**c**) Squared difference between the spectrum at t_start_ and t_end_ in the absence (black) or the presence of different APP concentrations: 5 µg/mL (pink), 10 µg/mL (yellow), 20 µg/mL (green), and 40 µg/mL (red).

We used circular dichroism (CD) to monitor the secondary structure variation of κ-casein incubated under amyloid aggregation conditions in the absence and in the presence of different APP concentrations. In Figure 4a, the κ-casein spectra at the beginning of the aggregation process, which starts with thermal incubation at 37 °C (t_0_), and after about 24 h (t_end_), corresponding to the plateau in protein fibrillogenesis kinetics (Figure 1), are reported. We found that, similar to what was reported by Farrell et al. [40], the protein during amyloid formation underwent a conformational transition with two spectral changes, one at 200 nm, and the other, more marked, at around 220 nm. As shown by electron microscopy experiments, these spectral changes are associated, by Farrell et al., with the formation of structures with fibrillar morphology. The fibrillization of κ-casein starts from the dissociation from the oligomeric state, which is considered the rate limiting step in the protein fibril formation [41,42]. The dissociated species, characterized by a significant percentage of β-native structure, would be the amyloidogenic precursor of fibrillar assembly through a β-sheet stacking that would not dramatically increase the total β-structure content but would be responsible for variations in the distribution of secondary β-strand and turn elements, thus determining the shape changes in the CD spectrum. These variations were not detectable when the protein was incubated with extracts from apple peel at 40 µg/mL polyphenolic content (Figure 4b), which was the most effective in preventing ThT fluorescence increase. The two spectra, at the beginning (t_start_) and after 24 h (t_end_) of incubation, almost overlap. At the APP concentrations’ intermediate range (5, 10, and 20 µg/mL), the higher the polyphenol content (Supplementary Materials, Figure S3), the more evident the changes in the spectra between that recorded at initial time t_start_ and after 24 h (t_end_) of incubation. To better visualize these results, we reported, in Figure 4c, the difference squared between the κ-casein spectra recorded at t_start_ and t_end_ in the wavelength region around 222 nm. As the figure shows, the extract from apple peel was able, in a concentration-dependent manner, to reduce the spectra changes sensitive to the conformational transitions that accompany the κ-casein amyloid formation.

### 2.5. Apple Peel Polyphenols Influence on κ-Casein Aggregation Monitored by Light Scattering

To further evaluate the κ-casein aggregation process, we carried out static and dynamic light scattering measurements of κ-casein incubated at 37 °C in the absence and in the presence of APP at the concentration of 40 µg/mL that, as revealed by ThT assay and CD measurements, was the most effective concentration among the tested ones in inhibiting amyloid formation. The light-scattering intensity depends on the molecular mass of a species in solution and, therefore, is a suitable technique to monitor the aggregation process. κ-casein is initially in an oligomeric micelle-like state, and this is reflected in the light-scattering intensity value that is detected at the beginning of the process.

**Figure 5:**
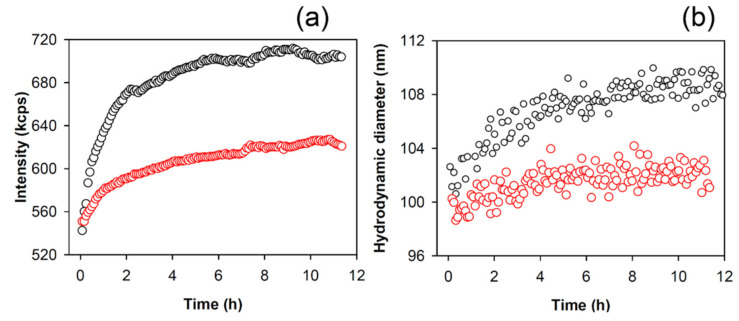
(**a**) The time course of the light scattered intensity (measured as kilo count per seconds (kcps), referring to the average number of photons per second arriving at the detector) from κ-casein (50 µM = 1 mg/mL) incubated at 37 °C in the absence (black circles) and presence of 40 µg/mL of apple peel polyphenols (red circles). (**b**) Time-course of the hydrodynamic diameters of κ-casein (50 µM = 1 mg/mL) incubated in the absence (black circles) and presence of 40 µg/mL of apple polyphenols (red circles) obtained from the cumulant analysis of the intensity autocorrelation function.

When κ-casein was incubated at 37 °C, an increase in the intensity scattered from the particles in solution occurred in time. The static scattered intensity, in particular, switched from 540 kcps to 700 kcps, from the beginning to the end of the process, with an increase of 30% indicating the occurrence of an aggregation process (Figure 5a). By the cumulant analysis of the intensity autocorrelation function generated by LS in dynamic modality, a correspondent increase in the average hydrodynamic diameter of species in solution from 100 to 110 nm was also found (Figure 5b).

When κ-casein was incubated with 40 µg/mL of APP extract, the increase in the scattered intensity was significantly reduced (Figure 5a). It switched from 540 to 610 kcps with an increase of only 13% compared to the κ-casein alone condition. A slight variation in the average hydrodynamic diameter of species in solution was observed as it remained at about 100 nm (Figure 5b).

### 2.6. Morphology of κ-Casein Aggregates Incubated with Apple Peel Polyphenols

**Figure 6:**
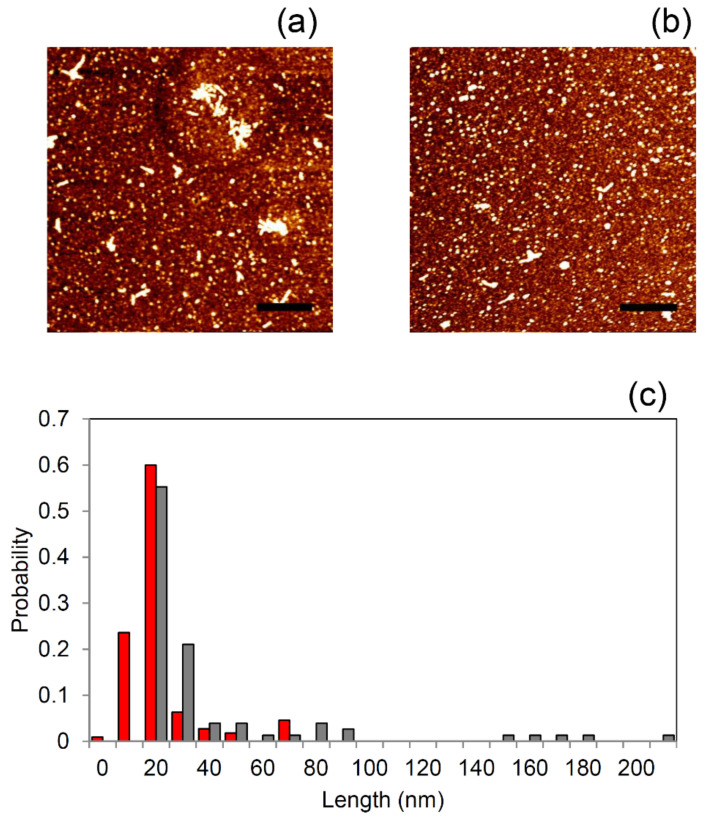
The morphology of κ-casein species formed in the absence and presence of polyphenols (**a**) κ-casein aggregates (50 µM) formed at the end of the aggregation kinetics (12 h). (**b**) κ-casein aggregates (50 µM) formed after 12 h in the presence of 40 µg/mL apple polyphenols. Scale bar = 400 nm and z-range = 5.3 nm. (**c**) Distributions of the lengths for the sample populations in the absence (grey) and presence of polyphenols (red).

To analyze the morphology of the κ-casein species formed at the end of kinetics in the presence or absence of 40 µg/mL APP, AFM imaging was applied. When κ-casein was incubated alone, fibrillar rod-shaped structures with average height of 5.13 nm and length of 100 nm (Figure 6a) were observed. According to the other techniques, when κ-casein was incubated in the presence of APP extract (40 µg/mL) (Figure 6b), the number of fibers and amyloid aggregates was found to be significantly reduced (Figure 6c).

### 2.7. Apple Peel Polyphenols Effect on Aβ_1–42_ Peptide Aggregation Monitored by Light Scattering

**Figure 7:**
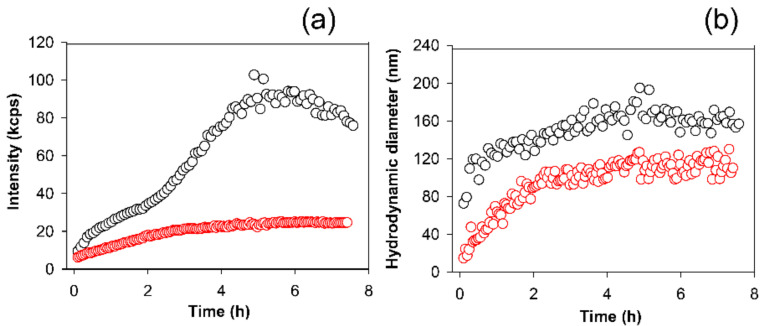
(**a**) Time-course of the light scattered intensity (measured as kilo count per seconds (kcps), referring to the average number of photons per second arriving at the detector) from 50 µM Aβ_1–42_ incubated at 37 °C in the absence (black circles) and presence of 40 µg/mL of apple peel polyphenols (red circles). (**b**) Time-course of the hydrodynamic diameters of Aβ_1–42_ incubated in the absence (black circles) and presence of 40 µg/mL of apple polyphenols (red circles) obtained from the cumulant analysis of the intensity autocorrelation function.

To add value to the results thus far obtained, we concluded our study by obtaining information on APP action on the amyloid aggregation process of a protein that, differently from κ-casein, is involved in a severe and common disease for human health. Hence, we tested the APP effect on the Aβ_1–42_ peptide involved in Alzheimer’ Disease by using the free-dye light scattering technique. It is known that heating from 0 to 37 °C induces, in the Aβ_1–42_ peptide, a conversion from coil to beta-strand structures typical of amyloid formation [69]. As shown in Figure 7a, when the Aβ_1–42_ peptide is incubated at 37 °C in the presence of APP, the increase found in the light scattered for the Aβ_1–42_ peptide alone, and indicative of the aggregation process, was strongly reduced.

However, as shown by the analysis of the hydrodynamic radii, also in this case, in the presence of APP, Aβ_1–42_ forms aggregated species, although lower in size with respect to the control (Figure 7b). Further studies should aim to better understand the structural and pathological features of the Aβ_1–42_ peptide species resulting from the APP; however, this is out of the objectives of the present study. We just want to highlight the importance of considering the synergic action of phenols from natural sources in the amyloid aggregation mechanisms at the basis of dangerous conditions for human health.

## 3. Discussion

The aim of our study was to gain insight into the ability of polyphenolic extracts to prevent the amyloid aggregation of the κ-casein, used as amyloidogenic protein model. The extracts were from apple peels, which are normally disposed of as waste in agriculture and the food industry. Several studies reported the ability of specific single polyphenols to inhibit both oxidative stress and protein aggregation associated with neurodegenerative diseases. However, few studies focused on the direct action on amyloid aggregation pathways exerted by the whole polyphenolic extract from fruit tissues generally considered as waste.

Research has shown that apples are a great source of bioactive compounds [5] with marked antioxidant activity and antiproliferative activity [5,6,7], and we demonstrated that the peels from the Fuji apples under study presented higher phenolic contents and more marked antioxidant activity with respect to the flesh and whole fruit (Table 1). The polyphenolic profile of Fuji apple peel extracts was studied using UHPLC–HESI–MS techniques, and seven phenolic compounds were identified (Supplementary Materials, Figure S1). In agreement with the literature data, a qualitative profile found vanillin, gentisic acid, sinapinic acid, ferulic acid, epicatechin, quercetin, and procyanidin A2, many of which have been revealed to be effective in preventing protein amyloid aggregation when singly tested [26,27,28,29,30,31,32,33], but they have never been tested in a natural phenolic pool.

To monitor the APP effect on amyloid formation mechanisms, we used κ-casein from bovine milk, a suitable model protein to study the aggregation processes in vitro. First, κ-casein forms fibrils at pH and temperature conditions (pH 7.0 and 37 °C) that well reproduce the physiological cell environment [41,42]. In addition, according to the Ecroyd model, a κ-casein monomer contributes more than one β-strand to the fibril structure, in a similar manner to that shown for the amyloid Aβ peptide involved in AD [70]. Similar to the peptides and proteins involved in several amyloid diseases (e.g., Alzheimer’s and Parkinson’s disease), κ-casein adopts an essentially unstructured conformation in its normal biological state (i.e., it belongs to the class of Intrinsically Disordered Proteins (IDPs)) [43,51]. More importantly, κ-casein forms micelles, and several studies hypothesized the crucial role of potential intermediate micellar conformations in amyloid fibrillogenesis [52,53,54].

Here, we show that APP at concentrations not toxic for cells (Figure S2) significantly inhibited κ-casein amyloid fibril formation in a concentration-dependent manner. Both the rate and the plateau of the protein fibrillogenesis kinetics profile were reduced in the presence of APP, and the effect was more marked at increasing concentrations of incubated polyphenols (Figure 1). At the highest phenol concentration tested (40 µg/mL), the thioflavin T increase appeared to be totally inhibited. The marked increase in ThT fluorescence results from the selective immobilization in the conformational ensemble of the dye. When free, the benzylamine and benzathiole rings of ThT can rotate with no constraints about their shared carbon–carbon bond. This rotation rapidly quenches the excited states generated by photon excitation causing low fluorescence emission for free ThT. Instead, in the presence of amyloid fibrils, the rings are sterically immobilized, and this preserves the excited state, resulting in a high fluorescence quantum yield. Therefore, ThT can be considered as a “molecular rotor” [71]. To exclude the potentially occurring interactions of polyphenols-ThT [35,67], we verified that fluorescence of the dye was not influenced by APP. In order to do this, we added 40 µg/mL APP to a sample of preformed κ-casein fibrils and ThT and verified that the dye fluorescence, enhanced by the presence of fibrillar structures, did not change in time (Figure 2), thus excluding the occurring of fluorescence quenching by the introduction of phenols.

Furthermore, we also used alternative dyes and dye-free tools to confirm the APP inhibitory properties. First, we confirmed the β-sheet formation in κ-casein incubated at 37 °C by using Congo red (CR) whose spectral shift assay is an alternative to ThT fluorescence for quantifying amyloid fibril formation [35]. The spectral shift present when CR is added to κ-casein after 24 h at 37 °C is absent when the dye is added to the protein incubated, under the same conditions, with APP (Figure 3). Then, we used circular dichroism to evaluate the APP ability to prevent the secondary structure variation that accompanies κ-casein amyloid formation. In agreement with thioflavin T experiments, this secondary structure variation was lower for higher APP concentrations, and the κ-casein spectrum registered after 24 h of incubation at 37 °C in the presence of APP at 40 µg/mL did not present significant changes in respect to the κ-casein at the initial state (Figure 4).

When κ-casein was incubated with APP at 40 µg/mL, the light scattered intensity variation from the sample was significantly reduced when compared to the control sample (Figure 5a) with very little variation in the average hydrodynamic diameter of species in solution observed over time (Figure 5b). The presence of aggregated species formed from κ-casein in the presence of 40 µg/mL APP, even if lower in size, morphologically different, and reduced in number with respect to the control, was also observed by AFM (Figure 6).

This is a very critical point because, as well established, amyloid oligomers smaller in size than the mature fibrillary structures are greatly more toxic than fibrils, due to a greater number of open active ends and their ability to diffuse in tissues [20,21,22]. However, the κ-casein aggregates formed in the presence of APP do not possess the amyloid cross-β spine structure that, in restricting the thioflavin rings rotation, determines a high fluorescence quantum yield. CD experiments further prove that these aggregates are prevented from forming the β-sheet stacking, which is responsible for the secondary structure content variation observed during κ-casein amyloid formation.

According to the model proposed by Ecroyd et al. [41,42], the κ-casein dissociation from the oligomeric state is the rate-limiting step in κ-casein fibril formation. The dissociated species, characterized by a significant percentage of β-native structure, would be an amyloidogenic precursor of fibrillar assembly through β-sheet stacking that would not dramatically increase the total β-structure content but would be responsible for variations in the distribution of secondary β-strands and turn elements. We hypothesize that this reorganization, leading to β-sheet packing occurring inside the first oligomers, is prevented by the interference with polyphenols.

**Figure 8:**
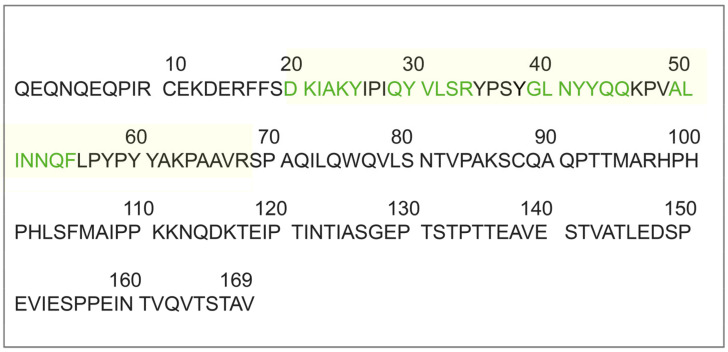
The amino acid sequence (signal sequence-deprived) of κ-casein from bovine milk (UniProtKB-P02668). The region involved in protein packing during amyloid formation is in light yellow. Residues belonging to native β-sheets are in green.

Research reported that polyphenols are capable of directly interacting with the protein amyloidogenic core through specific structural constraints and aromatic interactions between the phenolic compounds and aromatic residues in the amyloidogenic sequence. The packing in κ-casein amyloid formation would involve residues from position to 20 to 68 that contain not-stranded, highly hydrophobic β-sheets (from 20 to 25, 29 to 34, 39 to 45, and 49 to 55) connected by γ or β-turns [50]. These segments belong to a tyrosine-rich region (Figure 8), which, therefore, is highly susceptible to aromatic interactions with APP that we believe to occur before potential further packing leading to the amyloid core formation.

The aromatic interactions could be at the basis of the preventive action exerted by tannin and (−)-epigallocatechin-3-Gallate (EGCG) against κ-casein aggregation and, in general, for polyphenol inhibitors in amyloid aggregation through direct interactions with the proteins involved [28,34,35].

Our studies also revealed that APP did not show disrupter activity on κ-casein fibrils. This could be indicative of fibrillary stiffness or most likely, since only few protein segments are involved in the formation of the cross-β-sheet fibrillar structure, the remainder of the molecule could prevent the APP from reaching the fibril core, thus, impeding the formation of the preferential noncovalent interactions responsible for the dissolution of the fibrils.

## 4. Materials and Methods

### 4.1. Materials

κ-casein (UniProtKB-P02668) from bovine milk and all the reagents and chemicals were purchased from Sigma-Aldrich (St. Louis, MO, USA) except when mentioned specifically.

### 4.2. Plant Materials

Fuji apples were harvested at commercial maturity from “Scannale s.r.l.”, an organic farm located in Caltavuturo (37°49′ N and 850 m a.s.l.) (Sicily, Italy). Fruits were harvested at optimum maturity assessed by the starch iodine test (starch index value of 6; [72]). The apples were washed and stored at 4 °C.

Weight, longitudinal (LD) and transverse (TD) diameters, and flesh firmness of apples were detected. The fruits were individually weighted with a precision balance. LD and TD were measured with a digital caliper. The flesh firmness was measured on half of fruits with a penetrometer with a cylindrical 8 mm head (EFFEGI texture analyzer). Chemical parameters such as titratable acidity (TA), pH, and soluble solid content (SSC) were also detected. TA was performed by titrating an aliquot of 5 mL of filtered apple juice with a solution of NaOH 0.1 N and expressed in percentage of malic acid. The SSC were measured with digital refractometer (Atago) and reported as °Brix. Ripe Index (RI) was also calculated as the ratio between SSC, expressed in °Brix, and TA, and expressed in percentage (%). Physical parameters data: weight = (158.76 ± 0.45) g; LD = (60.10 ± 0.29) mm; TD = (66.70 ± 0.17) mm; firmness = (4.46 ± 0.45) Kg/cm^2^. Chemical parameters data: pH = 3.9 ± 0.1; TA = (0.30 ± 0.03) % malic ac.; SSC = 18.52 ± 0.10 °Brix; RI= 61.73 ± 0.83. All parameters were obtained by a mean of five measures. Thirty Fuji apples free of defects were selected and peeled (~1 mm thickness) then the apple peels were pooled and frozen in liquid nitrogen and stored at −80 °C until analysis.

### 4.3. APP Extraction, Phenolic Content Determination and Antioxidant Properties Evaluation

#### 4.3.1. Polyphenol Extraction

Polyphenols were extracted by a peel pool of at least thirty Fuji apples according to the method described by Ceymann et al. [73], with some modifications. Frozen peels were ground to fine powder by using a grinder and then the polyphenolic extraction was carried out. Briefly, an aliquot of the powder (2.50 g) was mixed with 50 mL of methanol containing 1% formic acid (*v*/*v*), homogenized for 15 min, and centrifuged at 10,000 rpm for 10 min. The supernatant was collected and filtered through 0.45 µm nylon filters. The polyphenols’ extraction was repeated three times. The same protocol was used to extract polyphenols from whole and flesh Fuji apple and compare the results with those obtained for the peel. For toxicity and k-casein experiments, the extraction solvent was removed by rotatory evaporator at 30 °C and the concentrated peel polyphenolic extract was dissolved in water, aliquoted, and kept at −20°C until analysis, avoiding direct contact with light and oxygen.

#### 4.3.2. Total Polyphenol Content (TPC) by Folin–Ciocalteu

The TPC analysis was performed following the polyphenols extraction by the Folin–Ciocalteu method in Ceymann et al. [73] with slight modifications. Two microliters of the Folin–Ciocalteu reagent and 2 mL of distilled water were automatically pipetted to 0.2 mL of the methanolic extract. After 1 min, 0.8 mL sodium carbonate solution (20%) and 0.8 mL distilled water were added, thoroughly mixed, and incubated for 30 min at 37 °C. Absorption was measured automatically at 750 nm. Total polyphenolic content was calculated with the external standard catechin calibration curve and expressed as mg catechin equivalents (CTE) per 100 g fresh weight (FW). The test was carried out in triplicate.

#### 4.3.3. Identification of Polyphenols by UHPLC–HESI-MS

Identification of polyphenols in Fuji apple peel extract was based on a reported procedure [74,75]. Phenolic compounds were identified by ultra-high-performance liquid chromatography, heated electrospray coupled with high-resolution mass spectrometry (UHPLC-HESI-MS) analysis using a quadrupole Orbitrap mass spectrometer (Thermo Fisher Scientific, Bremen, Germany). UHPLC analysis was performed using a Dionex Ultimate 3000 System (Dionex Softron GmbH, Germering, Germany) equipped with an auto sampler controlled by Chromeleon 7.2 Software (Thermo Fisher Scientific, Bremen, Germany). The column was a Phenomenex Luna C18(2) 50 × 1 mm, packed with core–shell particles of 2.5 μm. The flow rate was set at 50 μL min^−1^ at 20 °C and the total chromatographic analysis time was 52 min. The eluent A was water with 0.1% formic acid (*v*/*v*) pH 3.2, and eluent B was acetonitrile with 0.1% formic acid (*v*/*v*). The elution gradient program was 0–5 min 10% B; 5–45 min linear increase to 99% B, 45–50 min 10% B coming back to the initial conditions until full stabilization. The MS detection was conducted in two acquisition modes: full scan (negative ion mode) and targeted selected ion monitoring. For targeted selected ion monitoring analyses, a mass inclusion list containing exact masses of target phenolic compounds was built and applied.

#### 4.3.4. Oxygen Radical Absorbance Capacity (ORAC) Assay

The method reported by Cao et al. [76] was slightly modified and applied. The reaction mixture was prepared in a 96-well black microplate as follows: 160 μL of 0.04 μM Fluorescein in 0.075 M Na-K phosphate buffer pH 7.0, 20 μL of appropriately diluted extract or 20 μL of 100 μM Trolox used as control standard, or 20 μL of extraction solvent as blank. Each mixture was kept 10 min at 37 °C in the dark, and the reaction was started with the addition of 20 μL of 40 mM 2,2′-azobis (2-methylpropionamidine) dihydrochloride (AAPH). The fluorescence decay was measured at 37 °C every 1 min at 485 nm excitation and 538 nm emission, using a Thermo Scientific Fluoroskan Ascent F2 Microplate. The ORAC value refers to the net area under the curve (AUC) of fluorescein decay in the presence of ginger extract or Trolox, subtracted of the blank area. The activity of the sample was expressed as μmol of Trolox Equivalents (TE)/g of FW, with the following equation (Equation (1)):

ORAC value (μmol TE/g FW) = k × a × h × [(S_sample_ − S_blank_)/(S_Trolox_ − S_blank_)] (1)

where k is the extract dilution; a is the ratio between the volume (liters) of the extract and grams of sample used for the extraction; h is the final concentration of Trolox expressed as μmol/L; and S is the area under the curve of fluorescein in the presence of sample, Trolox, or blank.

### 4.4. Cell Culture and Cytotoxicity Assay

Mouse embryonic fibroblast cell line NIH-3T3 (Sigma-Aldrich) was cultured in Dulbecco’s Modified Eagle Medium (DMEM)-high glucose supplemented with 100 U/mL penicillin, 100 μg/mL streptomycin, and 10% bovine calf serum at 37 °C in 5% CO_2_ humidified atmosphere. NIH-3T3 cells were seeded into 96-well plates at a density of 104 cells per well in 100 μL of growth medium, cultured for 24 h, and then treated Fuji apple peel polyphenolic extract at final concentrations of 80, 40, 20, 10, or 5 μg/mL. All treatments were performed for 24 and 48 h of incubation in triplicate. Cell viability was analyzed by the CellTiter 96^®^ AQueous One Solution Cell Proliferation Assay (MTS assay, Promega, Milano, Italy) following the manufacturer’s instruction. In brief, after cell treatments, 20 μL of the MTS solution was added to each well and incubated with cells for 3 h at 37 °C, 5% CO_2_. The absorbance was read at 490 nm with the Bio-Rad iMarktm Microplate Reader (Promega, Milano, Italy). Cell viability was quantified as the percentage of viable cells using untreated cells as a control. All the experiments were repeated three times.

### 4.5. κ-Casein Amyloid Formation

A fresh stock solution of κ-casein in 50 mM phosphate buffer pH 7.4 was continuously stirred for 24 h and filtered through 0.22 μm filters before using. As verified by dynamic light scattering, this procedure assures sample homogeneity but not reduction to monomeric species, which cannot be obtained due to the presence, in the κ-casein sample, of stable self-associating oligomeric micelle-like species [40]. The protein concentration was determined by recording the absorbance spectrum after filtering the sample by using an extinction coefficient at 280 nm of 0.95 mg^−1^·mL·cm^−1^ [39] and considering the purity of the sample (70%) provided by Sigma Aldrich. κ-casein fibril formation is highly temperature-dependent [44]. To generate amyloid fibrils, all the experiments were performed incubating protein solutions at 50 μM without shaking at the mammalian physiological temperature of 37 °C.

### 4.6. Atomic Force Microscopy (AFM) Measurements

AFM measurements were performed by using a Nanowizard III (JPK Instruments, Berlin, Germany) mounted on an Axio Observer D1 (Carl Zeiss, Berlin, Germany) or on an Eclipse Ti (Nikon, Tokyo, Japan) inverted optical microscope. Aliquots of protein solutions were deposited onto freshly cleaved mica surfaces (Agar Scientific, Assing S.P.A., Monterotondo, Roma, Italy) and incubated for up to 20 min before rinsing with deionized water and drying under a low-pressure nitrogen flow. Imaging of the protein was carried out in intermittent contact mode in air by using NCHR silicon cantilever (Nanoworld, Neuchâtel, Switzerland) with nominal spring constant ranging from 21 to 78 N/m and typical resonance frequency ranging from 250 to 390 kHz.

### 4.7. Thioflavin T (ThT) Spectrofluorometric Measurements

ThT fluorescence emission was monitored by using a JASCO FP-6500 spectrometer (JASCO Corporation, Tokyo, Japan). The excitation and emission wavelengths were 450 and 485 nm, respectively, with a slit width of 3 nm. ThT concentration was 12 μM. κ-casein (50 μM) was dissolved with the dye before being placed at 37 °C in the thermostated cell compartment (10 mm). It is known that, upon binding to fibrils, ThT displays a dramatic shift of the excitation maximum (from 385 nm to 450 nm) and of the emission maximum (from 445 nm to 482 nm) [77]. Due to the binding of native κ-casein with Thioflavin T [40], data were presented as ΔF in function of time, where ΔF = F − F_0_ is the difference between the fluorescence reading value and F_0_ the fluorescence value registered once samples reached thermal equilibrium at 37 °C, after about 20 min.

### 4.8. Congo Red Binding Assay

The binding of Congo red was assessed by using absorption spectroscopy (Spectrophotometer Shimadzu UV2401PC, Tokyo, Japan). A 4 mM stock solution of Congo red (Sigma Aldrich) was prepared in 50 mM phosphate buffer pH 7.4. Congo red solution (20 µM) was mixed with κ-casein species (50 µM) taken at the beginning and after 24 h of protein incubation at 37 °C in the absence and in the presence of 40 µg/mL APP.

### 4.9. Aβ_1–42_ Peptide Amyloid Formation

The synthetic peptide Aβ_1–42_ (AnaSpec, Inc., Fremont, CA, USA) was solubilized in NaOH. 5 mM, pH 10, and lyophilized according to Fezoui et al. protocol [78]. The lyophilized peptide was then dissolved in 50 mM phosphate buffer pH 7.4 and filtered with two filters in series of 0.20 μm (Whatman, Merck KGaA, Darmstadt, Germany) and 0.02 μm (Millex-LG, Merck KGaA, Darmstadt, Germany) respectively, in order to eliminate large aggregates. The sample preparation was operated in asepsis using a cold room at 4°C. Aβ concentration was determined by tyrosine absorption at 276 nm using an extinction coefficient of 1390 cm^−1^·M^−1^. The aggregation kinetics of 50 µM Aβ_1–42_ was monitored by light scattering after incubating the sample at controlled temperature (37 °C) [69,79]. The samples containing 50 µM Aβ and APP were obtained by appropriate aseptic mixing of the protein solutions and placed in closed cuvettes in a cold room at 4 °C, before incubation at higher temperatures. The aggregation kinetics was followed, as for the control, at a controlled temperature (37 °C) and under controlled stirring (200 rpm) for 8 h.

### 4.10. Static and Dynamic Light Scattering

The aggregation of proteins in the presence or absence of polyphenols was investigated by static and dynamic light scattering [80,81]. The samples were placed into a dust-free quartz cell without further filtering and kept at 37 °C in the thermostatic cell compartment of a Brookhaven Instruments BI200-SM goniometer (Brookhaven Instruments, Holtsville, NY, USA). The temperature was controlled within 0.1 °C using a thermostatic recirculating bath. The light scattered intensity and its autocorrelation function were measured at θ = 90° by using a Brookhaven BI-9000 correlator (Brookhaven Instruments, Holtsville, NY, USA) and a 50 mW He-Ne laser tuned at a wavelength λ = 632.8 nm. Static light scattering data were corrected for the background scattering of the solvent and normalized by using toluene as a calibration liquid. The intensity I(q) is measured as kilo count per seconds kcps, referred to as average number of photons per second arriving at the detector.

Due to their Brownian motion, particles moving in solution give rise to fluctuations in the intensity of the scattered light. In a light scattering experiment carried out in dynamic modality, the autocorrelator measures the homodyne intensity–intensity correlation function that, for a Gaussian distribution of the intensity profile of the scattered light, is related to the electric field correlation function (Equation (2)):

(2) $$g^{\left(2\right)}\left(q,t\right)\;=\;{\left[A+Bg^{\left(1\right)}\left(q,t\right)\right]}^{2}$$

where *A* and *B* are the experimental baseline and the optical constant, respectively. For polydisperse particles, *g*^(1)^(*q*,*t*) is given by Equation (3):

(3) $$g^{\left(1\right)}\left(q,t\right)\;=\;\int_{0}^{\infty }G\left(\Gamma \right)exp\left(-\Gamma t\right)d\Gamma$$

Here, *G*(*Γ*) is the normalized number distribution function for the decay constant *Γ* = *q*^2^*D_T_*, where *q* = (4*π*n/λ)sin(θ/2) is the scattering vector defining the spatial resolution with n and *D_T_* being the solvent refractive index and the translational diffusion coefficient, respectively. The hydrodynamic diameter *D_H_* is calculated from *D_T_* through the Stokes–Einstein relationship (Equation (4)):

(4) $$D_{T}\;=\;\frac{k_{B}T}{3\pi \eta D_{H}}$$

where *k_B_* is the Boltzmann constant, T is the absolute temperature, and *η* is the solvent viscosity. *D_H_* was obtained by the intensity autocorrelation functions by means of the cumulant method [82].

### 4.11. Circular Dichroism

CD spectroscopic measurements were carried out at 20 °C by using a JASCO J-810 spectrometer (JASCO Corporation, Tokyo, Japan) equipped with a temperature control unit. A quartz cell with a path length of 0.2 mm was used for FAR-UV (190–250 nm) measurements. Samples of 50 μM κ-casein in the presence and in the absence of different APP concentrations were incubated at 37 °C. Aliquots of 100 μL were removed from each sample at the beginning and after 24 h of thermal incubation and quenched at 4 °C before CD experiments. Each CD spectrum was obtained by averaging over eight scans and subtracting the blank solvent contribution.

## 5. Conclusions

In this work, we demonstrated that the whole pool of polyphenols extracted from the peels of Fuji apple—a widespread apple variety—was effective in inhibiting the structural conversion accompanying the aggregation of κ-casein, a protein from bovine milk that forms amyloid fibrils under physiological conditions (neutral pH and 37 °C). Although more insights into the toxic properties of the protein species resulting from incubation with APP is required, we believe that our study lays the groundwork for considering a by-product of the apple food industry as a beneficial source for human health.

In fact, while the action of some individual polyphenols from apples on κ-casein and other amyloid proteins has been extensively proven, to our knowledge, the effect exerted by the pool of polyphenols extracted from apple peels (which, although rich in bioactive compounds, are normally considered as waste) has never been analyzed. Considering some common features between the Aβ peptides involved in Alzheimer’s Disease and κ-casein amyloid structures, including the cross-beta spine of the inner core [70] and the potential role of micellar conformations [52,53], we also verified that APP inhibit the aggregation of the Aβ_1–42_ peptide involved in Alzheimer’s Disease.

Therefore, our study could provide the basis for further research on the pathology of the protein species resulted from the APP inhibitory action thus favoring the valorization of agriculture and food industry waste products for recycling and circular economy purposes. The Fuji cultivar was introduced in Sicily only recently [83], and new knowledge on its functional properties could incentive this cultivation and help to preserve apple biodiversity.

More generally, our study further confirms the importance of designing new therapeutic effective strategies in the neurodegeneration field based on natural compounds and of investigating functional food consumption for prevention and health purposes.

## Figures and Tables

**Table 1:** The peel, flesh, and whole Fuji apple total phenolic content evaluated by Folin–Ciocalteu and the antioxidant properties assessed by ORAC assay. ORAC = oxygen radical absorbance capacity; TE = Trolox equivalents; FW = 100 g fresh weight; and CTE = catechin equivalents.

Tissue	ORAC Value (µmol TE/g FW)	TPC (mg CTE/100 g of FW)
Peel	118 ± 5	315 ± 20
Flesh	13 ± 5	42 ± 5
Whole fruit	22 ± 7	54 ± 8

## Data Availability

Data are contained within the article and Supplementary Materials.

## Supplementary Materials

The following are available online at. Figure S1: UHPLC–HESI-MS targeted single ion monitoring chromatogram obtained from Fuji apple peel phenolic extract, Figure S2: Effects of Fuji apple peel phenolic extract on NIH-3T3 cell viability. The cells were incubated for 24, 48, and 72 h with the appropriate concentrations of phenolic extracts. At the end of incubation, cell viability was measured by MTS assay as described in Materials and Methods. Values are means ± SD of cell viability calculated from at least three separate experiments, Figure S3: CD spectra of 50 µM κ-casein at the beginning of the amyloid aggregation process (black) and after 24 h of incubation at 37 °C (dashed black) in the presence of 5 µg/mL of APP (a); 10 µg/mL of APP (b); 20 µg/mL of APP (c).

## Sample Availability

Samples of APP are available from the authors.

## Abbreviations

APP	Apple Peel Polyphenols
CD	Circular Dichroism
LS	Light Scattering
ThT	Thioflavin T
TE	Trolox Equivalents
CTE	Catechin Equivalents
FW	Fresh Weight
AFM	Atomic Force Microscopy
FAR-UV	Far-UltraViolet
ORAC	Oxygen Radical Antioxidant Capacity
TPC	Total Polyphenol Content
CR	Congo Red
