# Supplementary material for: Valorization of Apple Peels through the Study of the Effects on the Amyloid Aggregation Process of κ-Casein
Source: Molecules. 2021 Apr 19;26(8):2371. doi: 10.3390/molecules26082371 (PMC8073991; doi:10.3390/molecules26082371)
Supplement: Supplementary file 1 [file molecules-26-02371-s001.zip › molecules-1154630-supplementary.pdf]

molecules

MDPI

# Supplementary Materials: Valorization of apple peels through the study of the effect on the amyloid aggregation process of κ-casein

**Valeria Guarrasi, Giacoma Cinzia Rappa, Maria Assunta Costa, Fabio Librizzi, Marco Raimondo, Vita Di Stefano, Maria Antonietta Germanà, Silvia Vilasi**

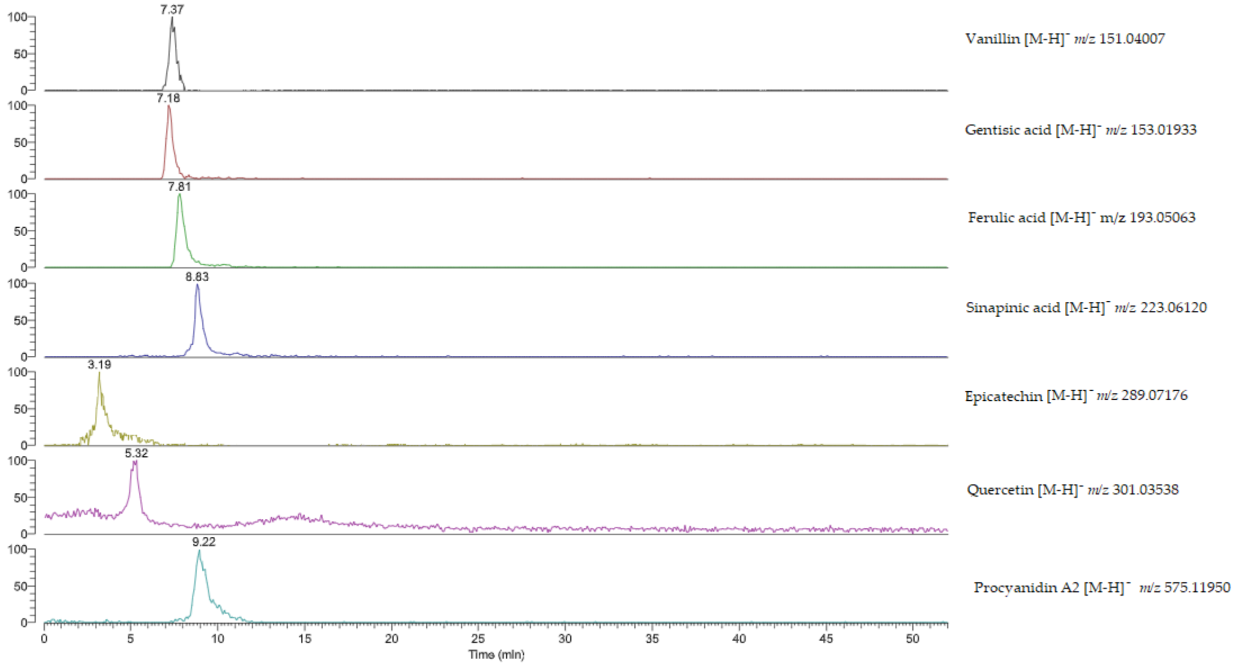


**Figure S1:** UHPLC–HESI-MS targeted single ion monitoring chromatogram obtained from Fuji apple peel phenolic extract.

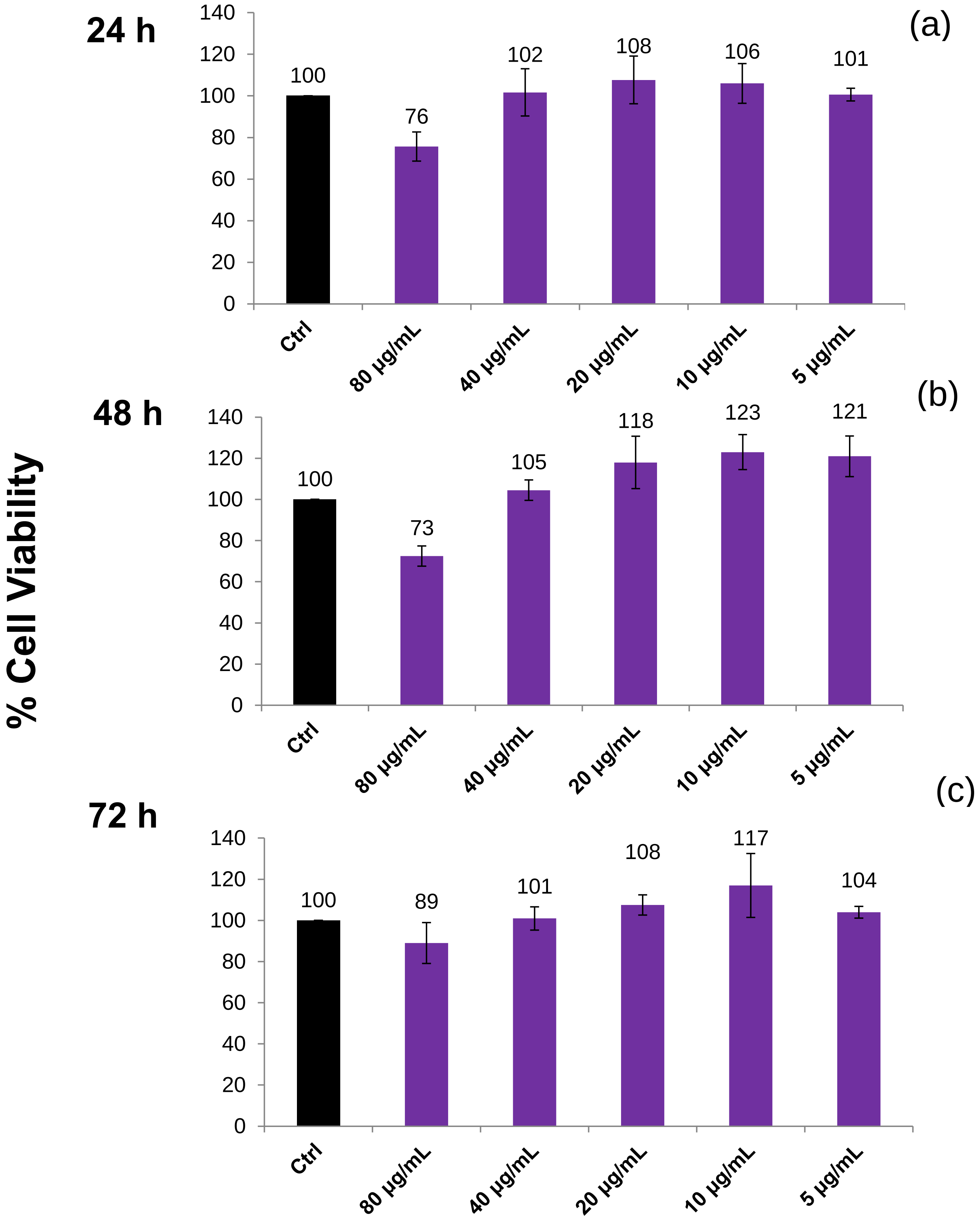


**Figure S2:** Effects of Fuji apple peel phenolic extract on NIH-3T3 cell viability. The cells were incubated for 24 h (a), 48 h (b), and 72 h (c) with the appropriate concentrations of phenolic extracts. At the end of incubation, cell viability was measured by MTS assay as described in Materials and Methods. Values are means ± SD of cell viability calculated from at least three separate experiments.

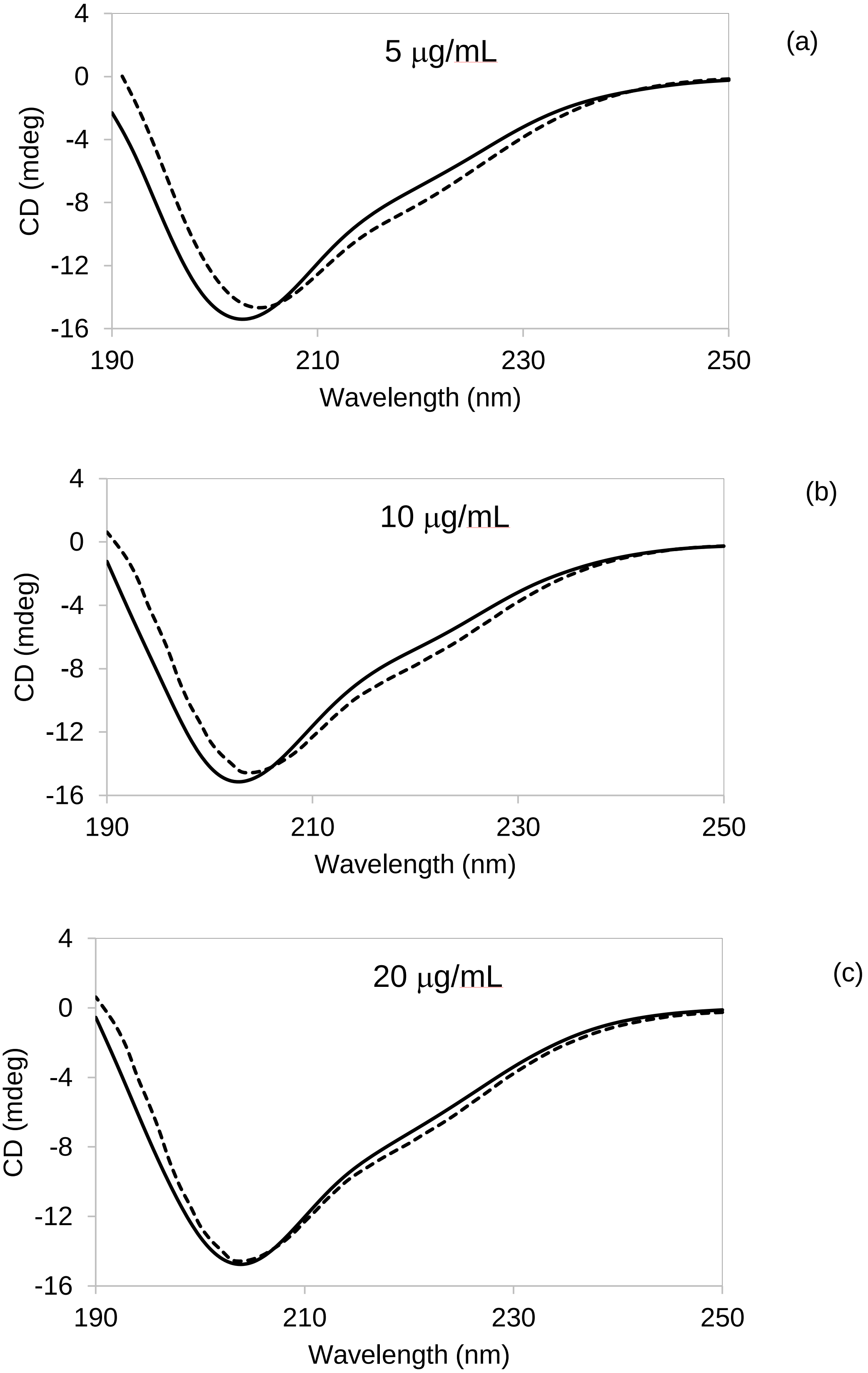


**Figure S3:** CD spectra of 50 μM κ-casein at the beginning of the amyloid aggregation process (black) and after 24 hours of incubation at 37 °C (dashed black) in the presence of 5 μg/mL of APP (a); 10 μg/mL of APP (b); 20 μg/mL of APP (c).
